# A computer-based matrix for rapid calculation of pulmonary hemodynamic parameters in congenital heart disease

**DOI:** 10.4103/1817-1737.53348

**Published:** 2009

**Authors:** Antonio Augusto Lopes, Rogério dos Anjos Miranda, Rilvani Cavalcante Gonçalves, Ana Maria Thomaz

**Affiliations:** Department of Pediatric Cardiology and Adult Congenital Heart Disease, Heart Institute (InCor), School of Medicine, University of São Paulo, São Paulo, Brazil

**Keywords:** Congenital heart disease, hemodynamics, oxygen consumption, pulmonary hypertension, pulmonary vascular resistance

## Abstract

**BACKGROUND:**

In patients with congenital heart disease undergoing cardiac catheterization for hemodynamic purposes, parameter estimation by the indirect Fick method using a single predicted value of oxygen consumption has been a matter of criticism.

**OBJECTIVE:**

We developed a computer-based routine for rapid estimation of replicate hemodynamic parameters using multiple predicted values of oxygen consumption.

**MATERIALS AND METHODS:**

Using Microsoft® Excel facilities, we constructed a matrix containing 5 models (equations) for prediction of oxygen consumption, and all additional formulas needed to obtain replicate estimates of hemodynamic parameters.

**RESULTS:**

By entering data from 65 patients with ventricular septal defects, aged 1 month to 8 years, it was possible to obtain multiple predictions for oxygen consumption, with clear between-age groups (*P* <.001) and between-methods (*P* <.001) differences. Using these predictions in the individual patient, it was possible to obtain the upper and lower limits of a likely range for any given parameter, which made estimation more realistic.

**CONCLUSION:**

The organized matrix allows for rapid obtainment of replicate parameter estimates, without error due to exhaustive calculations.

In children with congenital cardiac shunts, calculations of pulmonary and systemic blood flow and vascular resistance using the Fick principle involves many difficulties and sources of errors.[1] Probably, the most important concern is the use of assumed (predicted) values of oxygen consumption to calculate flow. Although measuring directly is preferable, predicting oxygen uptake is still a common practice in many institutions for several reasons.

A central problem with predicted oxygen consumption is that in general, one single formula for prediction is selected among several available ones.[2–6] As a result, it is not possible to know how different the predicted value is from the true oxygen uptake level in that specific patient. One possible solution is to obtain several predictions for oxygen consumption using different regression models (formulas). As a result, several values of pulmonary and systemic blood flow and vascular resistance will become available, and one can establish the upper and lower limits of a likely range for that patient. Although this sounds interesting, the procedure involves exhaustive calculations and the use of plenty of formulas, resulting in additional possibilities of errors.

Using Microsoft® Excel facilities, we developed a matrix containing all formulas needed to predict oxygen consumption by 5 different methods and calculate hemodynamic parameters taking each of the predictions into account. The general idea was to develop a method for obtaining multiple estimates of hemodynamic parameters at the same time (for example, pulmonary vascular resistance), thus allowing for the establishment of upper and lower limits of the likely ranges. By entering simple information such as demographics, hemoglobin concentration at the time of cardiac catheterization, intracardiac pressures and results of gas analyses, multiple results were quickly obtained without error due to exhaustive calculations.

## Materials and Methods

The first step consisted of preparing a computer-based matrix with all of the formulas required for calculations of oxygen consumption according to the 5 methods.[2–6] Except for the methods proposed by Bergstra *et al.*[5] and Wessel *et al.*,[2] where one single regression model (equation) is applicable to all patients, the tendency of other authors is towards using more than one equation.[3,4,6] Thus, our first task was to use 0 and 1 code columns to transform 2 or 3 equations (as is the case in the methods proposed by LaFarge and Miettinen,[3] Lindahl[4] and Lundell *et al.*[6]) into a single one. For example, while estimating oxygen consumption in a 2-year old child by the method proposed by Lundell *et al.*,[6] “codes 0” was used to eliminate the equations developed by the authors for males and females above the age of 3 years. Thus, the major work consisted of developing “single-equation models.”

The second step consisted of estimating oxygen consumption in 65 infants and children with ventricular septal defects, aged up to 8 years. For this purpose, demographic data were collected from unoperated patients without comorbidities or metabolic disturbances, in the absence of febrile or inflammatory states, and not using any medications that could potentially alter the heart rate. Patients were divided into 4 age groups, and oxygen consumption was estimated using the 5 predictive models for each age group. Statistical methods were applied to investigate within-group and between-group differences.

The third step consisted of extending the matrix to contain all the equations necessary for calculations of pulmonary and systemic arteriovenous differences in oxygen content, pulmonary and systemic blood flow and vascular resistance. Pulmonary and systemic flow and resistance were calculated for each estimated value of oxygen consumption. That is, the matrix was mounted so that the final results corresponded to the 5 estimates of pulmonary and systemic flow and flow ratios and pulmonary and systemic vascular resistance and resistance ratios.

### Statistics

Results of analysis of oxygen consumption in patients with ventricular septal defects are expressed as mean and standard deviation. Between-age group and within-age group differences were firstly analyzed by adjusting a general linear model to the obtained data. If differences were found to be significant, appropriate multicomparison tests were used to further investigate within-group (between methods) disparities. A significance level of 0.05 was adopted.

## Results

**Figure 1:**
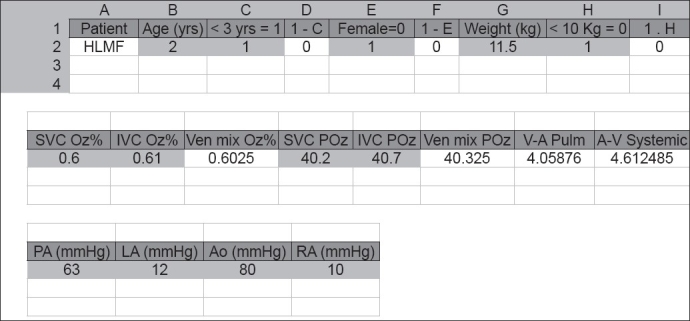
Example of data entry. Data such as age, sex, weight, height, heart rate, results of gas analyses, hemoglobin concentration and pressures within the cardiac chambers are provided by the user. Calculations such as body surface area and arteriovenous differences in oxygen content are performed automatically. Codes 0 and 1 are provided by the user to enable the system to select between 2 of 3 optional equations

**Figure 2:**
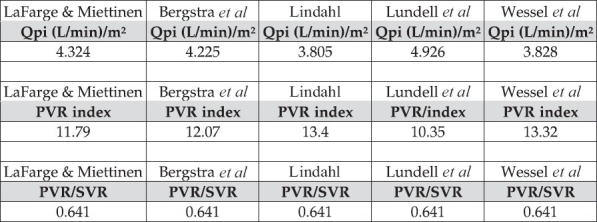
Example of output. For each patient, 5 estimates of pulmonary and systemic blood flow index (the former is represented as Qpi), pulmonary and systemic vascular resistance index (PVR index is expressed in Wood units.m^2^), as well as flow and resistance ratios (PVR/SVR), are rapidly calculated. Additional sets of results can be easily obtained by entering data collected in special conditions (e.g., during inhalation of nitric oxide, oxygen at high concentrations or administration of other pulmonary vasodilators)

Representative examples of data input and output are shown in Figures 1 and 2. By entering data from 65 children with ventricular septal defects (demographics and heart rate), oxygen consumption values predicted on the basis of 5 different formulas became immediately available. Mean values of predictions by the different methods (formulas) are shown in Table 1 for specific age groups (0–8 years). For each age group and every individual patient, it was possible to obtain several estimates of oxygen consumption, one of them (the lowest one) corresponding to the most pessimistic prediction of pulmonary vascular resistance (highest value) using the Fick principle. Thus, using a pre-established matrix containing the formulas, the between-groups and between-methods variability of predicted oxygen consumption could be investigated for a large number of patients, with the most impressive variability between methods being observed in the first age group [Table 1].

**Table 1:** Predicted oxygen consumption in children with ventricular septal defects

Age (years)	N	1	2	3	4	5
						
		LaFarge and Miettinen (ml/min)/m^2^	Bergstra *et al.* (ml/min)/m^2^	Lindahl (ml/min)/m^2^	Lundell *et al.* (ml/min)/m^2^	Wessel *et al.* (ml/min)/m^2^
0–2.0	28	198 ± 16	226 ± 48	156 ± 12	169 ± 24	164 ± 5
>2.0–4.0	9	165 ± 5	155 ± 8	156 ± 6	191 ± 17	155 ± 1
>4.0-6.0	12	147 ± 6	145 ± 6	151 ± 8	170 ± 13	152 ± 1
>6.0–8.0	16	145 ± 9	146 ± 6	147 ± 6	168 ± 15	151 ± 1

Results are expressed as mean and standard deviation. Differences between and within age groups were significant (*P* < .001, adjusted general linear model). For the age group of 0-2.0 years, the value obtained with method 1 was different from all the remaining ones, the same for method 2 (*P* < .01). Values obtained with methods 3, 4 and 5 were not different. For the other age groups, only the values provided by method 4 were significantly different (*P* < .01)

Next, we used the automatically calculated pulmonary and systemic arteriovenous differences in oxygen content, the mean pressures (pulmonary artery, left atrium, aorta and right atrium) and the 5 estimates of oxygen consumption to generate for the individual patient, 5 different predictions for pulmonary and systemic blood flow, pulmonary-to-systemic flow ratio, pulmonary and systemic vascular resistance and pulmonary-to-systemic resistance ratio. Table 2 shows 2 of the 5 instantaneously generated estimates of hemodynamic parameters in a representative patient, with data (demographics, heart rate, hemoglobin concentration, results of gas analyses and pressures within the cardiac chambers) collected under 2 conditions: Baseline and during nitric oxide inhalation.

**Table 2:** Upper and lower estimates of pulmonary vascular resistance and other parameters based on predicted oxygen consumption: Representative patient data (*)

	Baseline		40 ppm Nitric oride	
		
Parameter	Estimate 1	Estimate 2	Estimate 1	Estimate 2
Qpi (L/min)/m^2^	4.40	6.60	8.74	13.12
Qsi (L/min)/m^2^	2.26	3.39	2.40	3.60
Qp/Qs	1.95	1.95	3.65	3.65
PVRi (U. m^2^)	3.64	2.42	1.71	1.14
SVRi (U. m^2^)	19.91	13.27	16.69	11.12
PVR/SVR	0.18	0.18	0.10	0.10

(*) Data are from a 7-month-old infant with atrial and ventricular septal defects. Estimates 1 and 2 correspond, respectively, to values obtained using the formulas of Lindahl and Bergstra *et al.* PVRi and SVRi, are, respectively, pulmonary and systemic vascular resistance index; PVR/SVR is the pulmonary-to-systemic vascular resistance ratio; Qpi and Qsi are, respectively, pulmonary and systemic blood flow index; Qp/Qs is the pulmonary-to-systemic flow ratio

## Discussion

In this report, a computer-based matrix was developed for rapid calculations of hemodynamic parameters in patients with congenital heart disease subjected to cardiac catheterization. The main goal was to quickly obtain multiple estimates of several hemodynamic parameters without errors due to calculations.

The majority of formulas included in the matrix corresponded to regression models developed for prediction of oxygen consumption. Predicted values of oxygen uptake have been largely considered as unreliable.[7] Actually, in some instances, these values have been shown to be higher than the measured ones,[8] indicating that there was underestimation of pulmonary vascular resistance using the Fick principle. On the other hand, it is notorious that cyanotic patients have higher levels of oxygen uptake than expected on the basis of predictions. Based on these discrepancies, some specialists would prefer to rely on the pulmonary-to-systemic vascular resistance ratio (which does not require oxygen consumption to be calculated) for decision making. Less conservative specialists argue that mild variations in oxygen consumption are unlikely to have a significant impact on final calculations, and the need for an exact value of pulmonary vascular resistance may be overrated if all other clinical and noninvasive data are available.

By using a rapid-calculation method, we were able to obtain replicate estimates of the same parameter (e.g., oxygen consumption, pulmonary and systemic flow and vascular resistance) and establish the upper and lower limits of a likely range for a given patient. For example, for the case depicted in Table 2, the upper and lower estimates of pulmonary vascular resistance during inhalation of nitric oxide were 1.14 and 1.71 Wood units, respectively. That is, if that patient had univentricular physiology and was being considered for completion of cavopulmonary anastomoses (so-called Fontan operation, where precise preoperative measurements are needed), we would be relatively comfortable in terms of defining operability, since for him, the likely range was below 2.0 Wood units, which is considered as adequate for this type of operation.

Despite the facilities available while managing the prepared matrix, we remain concerned about the unrestricted use of predicted oxygen consumption.[1,7,8] This practice should be avoided in at least 3 situations: 1- cyanotic patients; 2- patients with comorbidities, metabolic disturbances or under the use of any drugs that might potentially affect the heart rate; 3-scenarios where very precise hemodynamic estimates are needed and the obtained ranges override acceptable limits.

In conclusion, in the vast majority of patients with congenital cardiac shunts associated with pulmonary hypertension, assignment to correction (surgical or catheter interventional) is based on a broad spectrum of information obtained from noninvasive evaluation. In a smaller group of patients, however, a clinical history of pulmonary congestion and failure to thrive is not present, and sometimes mild peripheral oxygen desaturation is associated with bidirectional shunting across the defect. Measurement of pulmonary vascular resistance is mandatory in these instances. If for any reasons pulmonary vascular resistance cannot be calculated on the basis of measured oxygen consumption, it is probably better to have estimates at the upper and lower limits of a likely range for that specific patient. The matrix proposed in this report has the advantage of allowing rapid calculations of multiple hemodynamic parameters using different values of predicted oxygen consumption, provided that the limitations of using predicted consumption are taken into consideration. Further studies are obviously necessary to evaluate the impact on outcomes of having intervals for hemodynamic parameter estimation instead of single values.

### Availability

For academic purposes, the prepared computer-based matrix can be obtained free of charge at aablopes@usp.br.
